# Effects of Void Characteristics on the Mechanical Properties of Carbon Fiber Reinforced Polyetheretherketone Composites: Micromechanical Modeling and Analysis

**DOI:** 10.3390/polym17131721

**Published:** 2025-06-20

**Authors:** Yong Zhang, Yibo Li, Xi Luan, Bin Meng, Jinsong Liu, Yan Lu

**Affiliations:** 1 College of Mechanical and Electrical Engineering, Central South University, Changsha 410083, China; 18004879141@163.com (Y.Z.); luyan2161@163.com (Y.L.); 2 Light Alloy Research Institute, Central South University, Changsha 410083, China; mengbin1219@126.com (B.M.); jinsongliu0108@163.com (J.L.); 3 Jiangsu Automation Research Institute (Shanghai), Shanghai 222006, China; drluanxi@126.com

**Keywords:** composite materials, representative volume element (RVE), void characteristics, mechanical properties, finite element analysis (FEA)

## Abstract

This study proposes a novel algorithm for generating representative volume elements which mitigate microstructural inhomogeneities in fiber-reinforced composites. The algorithm integrates void characteristics obtained from micro-computed tomography to more accurate microstructure models. Based on these models, the effects of void content, spatial distribution, and void diameter on the mechanical behavior of CF/PEEK composites are systematically evaluated using finite element analysis and experimental validation. The results reveal that void content significantly reduces transverse tensile strength and ductility, while void size further accelerates failure and enhances brittleness. In contrast, void distribution has minimal influence on the transverse mechanical response. These findings not only offer qualitative insights into void-induced damage mechanisms but also provide a theoretical basis for optimizing microstructures to enhance the mechanical performance of CF/PEEK and similar composite systems. Finally, the limitations of this study have been discussed, and directions for future research are proposed.

## 1. Introduction

With the increasing demand for lightweight and high-strength materials in the aerospace, automotive, and energy industries, fiber-reinforced composites have become crucial, owing to their excellent specific strength and stiffness [1,2,3]. The rapid expansion of composite material applications highlights the need for accurate failure prediction and prevention. Although experimental methods provide valuable insights into material performance, they are often time-consuming, labor-intensive, and costly [4,5,6]. Additionally, these methods typically lack precise control over microstructural features, making it challenging to assess the effects of internal defects on composite behavior. Representative volume element (RVE)-based modeling provides an effective approach for predicting the overall mechanical behavior of composites and serves as a valuable tool for their design and optimization [7,8]. Consequently, RVE-based micromechanical analysis is crucial for addressing the limitations of experimental techniques.

To effectively model the irregular spatial arrangement of fibers in composite microstructures, researchers have developed statistically equivalent RVEs. Previous studies have assumed a regular and periodic fiber arrangement to simplify the modeling process. However, these periodic models have significant limitations in capturing the randomness and variability in real composite microstructures. Trias et al. [9] compared stress and strain distributions under transverse tensile loading in periodic and realistic fiber arrangements and found that periodic models were inadequate for predicting damage and failure. Similarly, Brockenbrough et al. [10] demonstrated that periodic fiber arrangements could not accurately predict transverse deformation under plastic conditions. Gusev et al. [11] highlighted the significant influence of microstructural randomness on the transverse elastic constants of composites and the need to incorporate fiber distribution randomness into analyses of damage initiation and progression in the matrix. To address the limitations of earlier models, researchers have developed various algorithms to generate random fiber distributions and produce more realistic composite microstructure models. For example, Pyrz [12] introduced the hard-core model, while Oh et al. [13] developed an improved algorithm based on this approach. Although these methods have substantially increased the maximum achievable fiber volume fractions, they face difficulties in generating distributions with fiber volume fractions above 50%. This limitation poses a critical challenge for the current study, as fiber volume fractions typically reach or exceed 60%.

Experimental fiber distribution methods, such as the nearest-neighbor algorithm by Vaughan and McCarthy [14], can accurately capture short-range fiber interactions. However, these methods are time-consuming and costly owing to their dependence on specialized software and hardware. To overcome the volume fraction limitations of the hard-core model, Yang et al. [15] developed the RSE model, which enables fiber volume fractions exceeding 65%. However, this method faces challenges with fiber clustering in the central region, which reduces the uniformity of the composite microstructure.

Although various RVE-based models are widely used for fiber-reinforced composites [7,8,16,17], numerous studies have either neglected porosity or modeled its effects using simplified empirical models. While these methods provide preliminary insights into composite performance, recent research [18,19] has shown that empirical models alone cannot accurately predict composite behavior. Porosity reduces the effective material volume, disrupts stress transfer, induces local stress concentrations, promotes crack propagation, and contributes to other failure mechanisms [14,20,21,22]. Therefore, advanced analytical methods are urgently needed to elucidate the role of voids in composite materials.

The porosity levels of CF/PEEK fiber-reinforced composite laminates vary significantly depending on the molding process. Laminates produced by automated fiber placement (AFP) technology typically exhibit high porosity, which often exceeds 3.0% [23,24,25]. In contrast, laminates produced via autoclave, out-of-autoclave vacuum bag (OVB), or hot-press processes exhibit significantly lower porosity, which can be reduced to below 0.8% under optimized processing parameters [23,26]. Regarding void characterization, Almeida Jr et al. [27] employed segmented CT scan data to analyze void features in composite materials.

This study introduces a novel RVE generation algorithm for fiber-reinforced composites, which eliminates local structural inhomogeneities and incorporates pore features within RVE. This algorithm accurately predicts the macroscopic properties of the materials. Moreover, the effects of void characteristics—including content, spatial distribution, and diameter—on damage and failure mechanisms are investigated using appropriate constitutive models and failure criteria. The findings provide a theoretical basis for optimizing the performance and reliability of fiber-reinforced composites.

## 2. Statistical Analysis of CF/PEEK Porosity Characteristics

### 2.1. Material and Specimen Preparation

In this study, PEEK-CF60 prepreg tape supplied by Evonik (Germany) is used to prepare specimens. The tape features a monolayer thickness of 0.14 mm, an initial fiber volume fraction of 60%, and an initial void content (VC) of 2.34%. The tape has a melting temperature of 343 °C, with a recommended molding temperature range of 370–410 °C and a molding pressure of 1 ± 0.5 MPa. Detailed performance parameters of the prepreg tape are provided in Table 1.

**Figure 1:**
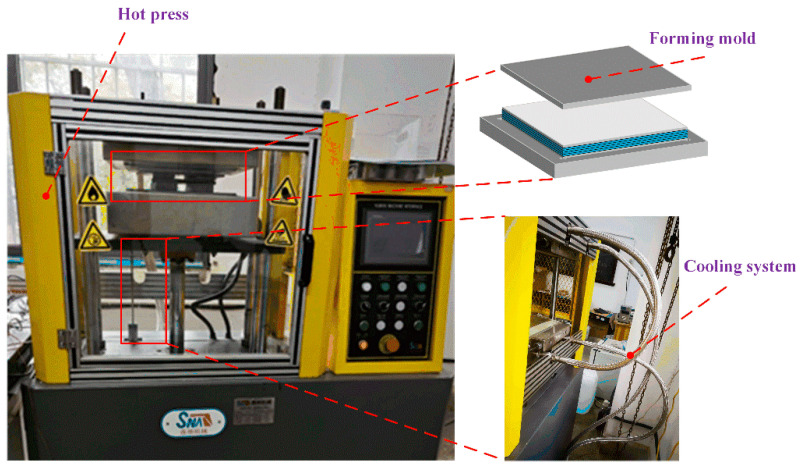
Schematic of the hot press molding system.

To achieve a distinct stepwise distribution of porosity in the fabricated samples, the molding temperature is set to the minimum recommended value of 370 °C. During sample preparation, three molding pressures—0.6, 1.0, and 1.4 MPa—are applied. The specimens are fabricated as follows: First, the prepreg tape is cut into 100 mm × 100 mm sheets and manually stacked into 16 layers, all aligned in the 0° fiber direction. The stacked prepreg is then placed in a mold. Finally, the specimens are thermoformed using a hot press molding system (10TYJ/B, Sena Machinery). Figure 1 shows a schematic of the hot press molding system.

The hot-pressing process involves three main stages:

(1) The hot press system is heated from room temperature to the molding temperature at a rate of 10 °C/min, with a pre-pressure of 0.1 MPa applied.
(2) Once the molding temperature is reached, the pressure is increased to the target molding pressure and maintained for 30 min to ensure proper consolidation and bonding of the prepreg layers.
(3) Finally, the system is cooled to room temperature under constant pressure to prevent defects and ensure dimensional stability.

### 2.2. Micro-Computed Tomography (Micro-CT) Scanning

To comprehensively analyze VC, void morphology, and distribution patterns in the composites, high-resolution micro-CT (Xradia 620 Versa, Carl Zeiss, Germany) was utilized. Test samples were extracted from three evenly spaced locations near the center of the laminate. Each sample measured 5 mm × 10 mm, with its length aligned along the fiber direction.

### 2.3. Extraction of Void Characteristics

**Figure 2:**
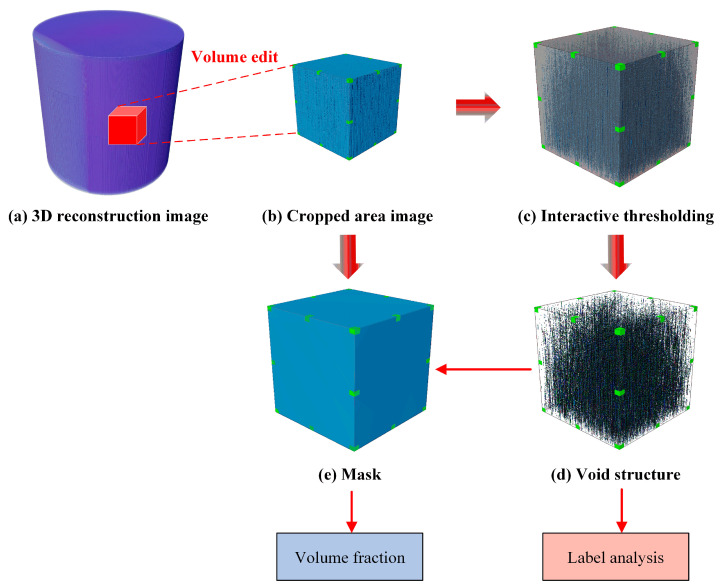
Image segmentation procedure for pore extraction in carbon fiber composites.

The original images obtained from μ-CT scanning consist of a series of parallel 2D microscopic slices and provide comprehensive 3D data about the entire sample volume. Each voxel is assigned a grayscale intensity corresponding to the material density. Material boundaries are automatically identified through grayscale analysis, in which voids (air) are represented as black and the material is visualized as white. Avizo 2020 is used for extensive analysis of the micro-CT images of the composite material. The analysis involves several key steps. Figure 2 illustrates the image segmentation procedure for void extraction in the composites.

(1) The raw data are processed and reconstructed into a detailed three-dimensional (3D) model.
(2) Core data within the targeted area are precisely cropped from the model to remove unnecessary portions.
(3) Grayscale values are carefully adjusted to define the contours and features of the voids, thereby improving recognition efficiency and analysis accuracy.
(4) Based on optimized grayscale range, thresholding and image segmentation are performed to accurately identify void regions. Through extensive comparative analysis, the optimal grayscale threshold range for accurate void identification is determined to be 0–4852 under 16-bit grayscale conditions.
(5) To address pore stacking issues, a watershed algorithm is applied to separate connected voids and extract detailed void morphologies.
(6) The label analysis tool is used to perform a thorough statistical evaluation of various void characteristics.
(7) Finally, VC is calculated as the ratio of the total void volume to the volume of the study area.

### 2.4. Quantitative Characterization of Pore Models

**Figure 3:**
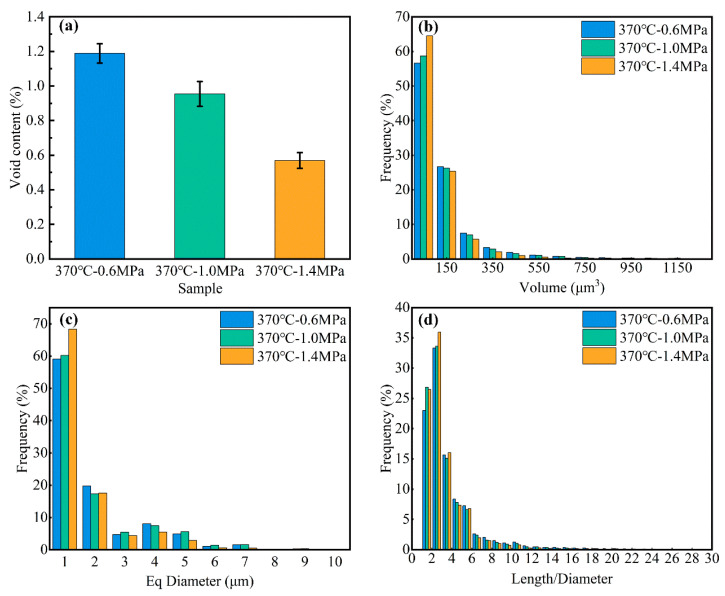
Three-dimensional analysis of sample voids: (**a**) VC; (**b**) void volume distribution; (**c**) void aspect ratio (length-to-diameter); (**d**) equivalent diameter distribution.

After the micro-CT data were processed with Avizo 2020, a detailed quantitative analysis of void characteristics was performed. Figure 3 presents statistical data on VC, void volume distribution, shape, and equivalent diameter under three molding pressures.

The VC in the composite materials gradually decreases with increasing molding pressure (Figure 3a). Specifically, the sample molded at 0.6 MPa exhibits the highest porosity (~1.2%), while the sample molded at 1.4 MPa features the lowest porosity. These results indicate that higher molding pressures effectively reduce void formation.

The statistical analysis of void volume distribution frequency (Figure 3b) reveals that despite differences in overall porosity among the samples, their void volume distribution patterns remain highly consistent. Most small-volume voids are concentrated in the 0–750 µm^3^ range. In contrast, larger-volume voids are comparatively rare.

Previous studies [28] have shown that void morphologies on the cross-sectional plane are approximately elliptical, as ellipses more accurately represent the true geometry of voids. Moreover, key attributes such as the mass center, volume, and moment of inertia remain consistent before and after void profile fitting. These findings indicate that elliptical voids can be further simplified as circles for analytical purposes. Consequently, this study characterizes void size on the cross-sectional plane using the equivalent diameter, calculated as follows [29,30]:

(1) $$D_{eq}=\sqrt{4A/\pi }$$

Figure 3c illustrates the frequency distribution of void equivalent diameters. Most voids have equivalent diameters concentrated within the 1–7 µm range, with larger voids being nearly absent. This observation is highly consistent with the findings reported in reference [31]. According to statistical analysis, selecting the 1–7 µm range as the representative void diameter effectively captures the dominant size distribution observed in the experiment. This selection ensures both scientific accuracy and representativeness for subsequent modeling. The corresponding frequency distribution of equivalent diameters is [0.6078, 0.1749, 0.0553, 0.0753, 0.0561, 0.0141, 0.0165], which accurately reflects the actual void size distribution obtained from the experimental data.

Although molding pressure effectively reduces the overall porosity, it has a minimal impact on void diameter distribution. This indicates that void size characteristics are mainly influenced by the material preparation process rather than molding pressure. To further assess void shape, the ratio of void length to equivalent diameter was calculated (Figure 3d). Statistical analysis reveals that voids with a ratio below 1 are nearly absent, while most values fall within the range of 2–20. These results suggest that the voids are predominantly elongated and cylindrical, with their lengths substantially exceeding their diameters.

According to these findings, voids are modeled as cylinders with diameters ranging from 1 to 7 µm. The experimentally derived frequency distribution of void diameters is incorporated into RVEs, ensuring that the constructed models accurately represent the actual void distribution within the material. This modeling approach provides a reliable basis for investigating the effect of void characteristics on the mechanical behavior of fiber-reinforced composite materials.

## 3. Generation of RVE Units with Embedded Pore Characteristics

The key challenges in establishing an RVE for thermoplastic composite materials include the following: (1) the difficulty of using conventional fiber generation algorithms to effectively model RVEs with fiber volume fractions exceeding 50%; (2) the need to incorporate voids with randomly varying sizes and spatial distributions; (3) the challenge of defining appropriate damage criteria to accurately simulate the mechanical behavior of composites, including elastic modulus, tensile strength, compressive strength, shear strength, and internal damage mechanisms.

**Figure 4:**
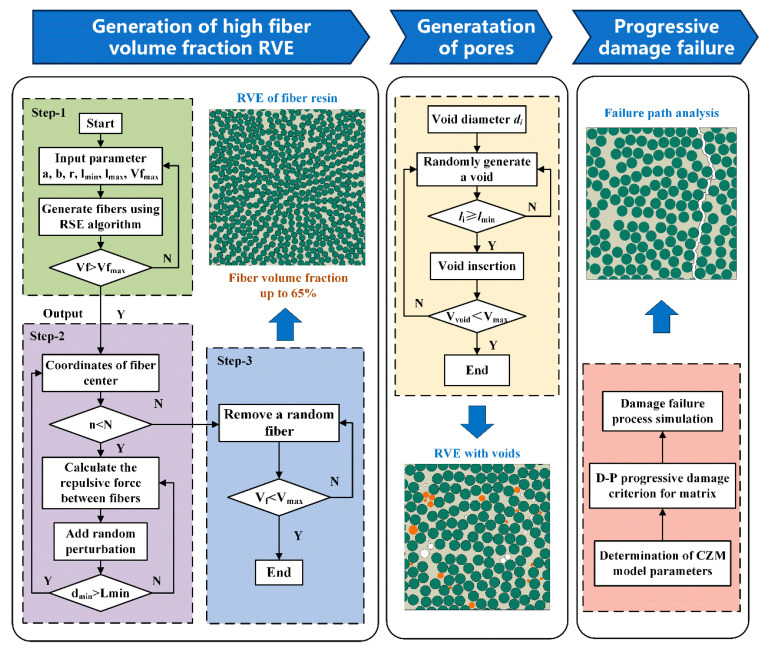
Workflow of the modeling methodology for analyzing void characteristics.

To address these challenges, this study first introduces a random sequential expansion–uniform distribution (RSE-UD) algorithm to generate RVEs containing both fibers and resins. Moreover, a probability density function-based method is developed to enable the random insertion of void features within RVEs. Finally, a mechanical modeling approach is established using the cohesive zone model and an extended Drucker–Prager progressive damage criterion. The overall modeling methodology and workflow are illustrated in Figure 4.

### 3.1. RVE Construction Method for Composites with High Fiber Volume Fraction

The RSE-UD algorithm involves the following steps:

(1) Initial fiber layout generation: Fibers are first generated based on the specified fiber volume fraction using the RSE algorithm.
(2) Simulation of particle interactions: A force model combined with a stochastic perturbation mechanism is applied to simulate fiber interactions, which gradually eliminate local structural irregularities.
(3) Fiber removal: Excess fibers are randomly removed to achieve the target fiber distribution and produce the final RVE.

**Figure 5:**
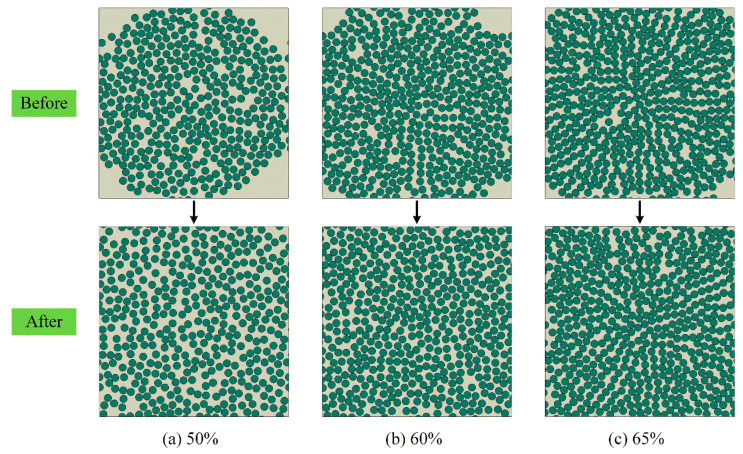
Comparison of RVEs before and after homogenization at different fiber volume fractions.

This algorithm enables the generation of RVEs with fiber volume fractions up to 67%, which exceeds the conventional 50% limit. Figure 5 compares the RVE generation results before and after the application of the improved algorithm at different fiber volume fractions. The results indicate that the new algorithm maintains the efficiency of the original RSE method and significantly improves fiber distribution uniformity through the incorporation of a gravity-based model.

To confirm the effectiveness of the proposed RSE-UD algorithm for generating randomly distributed fibers, the statistical equivalence of the resulting RVEs was evaluated from four key perspectives: (1) nearest neighbor distance, (2) nearest neighbor orientation, (3) Ripley’s K-function, and (4) pair distribution function.

**Figure 6:**
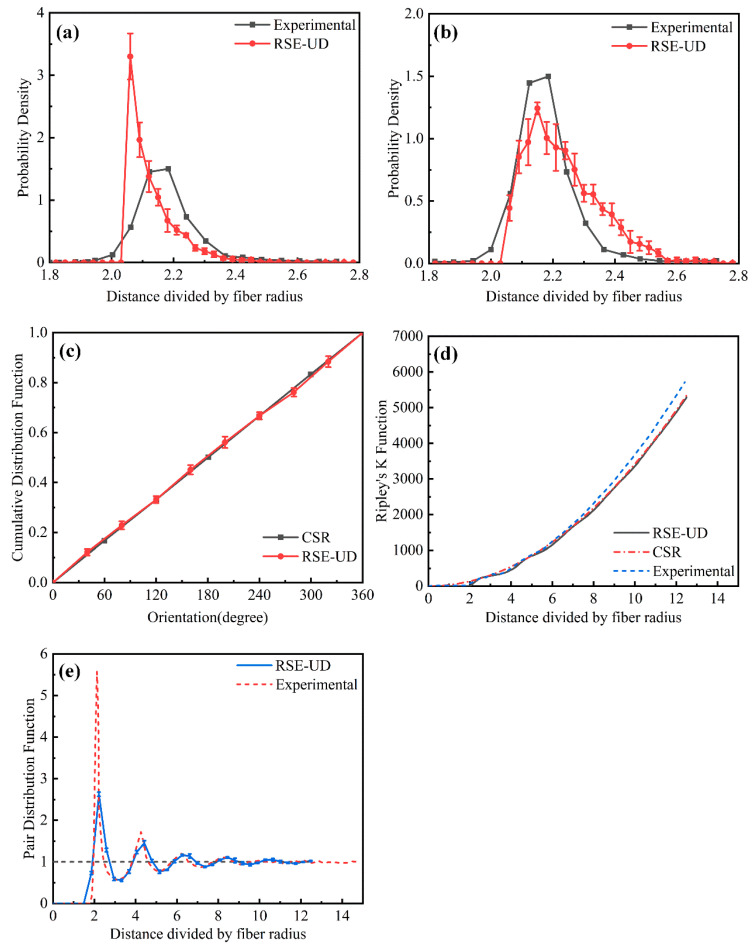
Statistical equivalence analysis of RVEs: (**a**) first nearest neighbor distribution function; (**b**) second nearest neighbor distribution function; (**c**) nearest neighbor orientation function; (**d**) Ripley’s K-function; (**e**) pair distribution function.

Figure 6 compares RVEs generated by the RSE-UD algorithm with experimental data from Reference [14] and results from the complete spatial randomness model to assess statistical equivalence. A comprehensive statistical analysis of 20 microstructural samples reveals that the random fiber distributions in RVEs produced by the RSE-UD algorithm closely match the actual material microstructures. Therefore, RVEs generated by this algorithm are highly suitable for micromechanical analysis.

To further validate the accuracy and reliability of the proposed RSE-UD algorithm, 20 randomly generated RVE models were used to predict the effective elastic properties of carbon fiber-reinforced composites. Table 2 presents the predicted average elastic constants from these RVEs and experimental results from Reference [21]. The model predictions exhibit a strong correlation with the experimental data, particularly for the longitudinal elastic properties. However, some deviations are observed in the transverse modulus (*E*_22_) and the in-plane shear modulus (*G*_12_). Numerous studies have reported similar discrepancies in transverse properties [21,32]. These differences are typically attributed to the presence of interphases, voids, and other internal defects in the actual material, which are not accounted for in the numerical model.

### 3.2. Void Distribution Model

According to the void diameter range and probability density distribution analyzed in Section 2, voids are incorporated into the fiber–resin composite to construct an RVE model. The generation algorithm proceeds as follows:

(1) The fiber–resin RVE serves as the base model, with the void diameter range and probability density distribution obtained from statistical analysis used as input parameters.
(2) Voids are randomly generated within the RVE domain according to the probability density distribution of void diameters. Each void is assigned a diameter and a random spatial position.
(3) The generated voids are checked for overlap or interference with existing fibers or other voids. If interference occurs, the voids are regenerated until all conditions are satisfied.
(4) The total porosity of the generated voids is calculated. If the porosity does not meet the target value, steps 2 and 3 are repeated until the desired porosity is achieved, thereby completing the generation process.

A void distribution consistent with the statistical characteristics is introduced into the fiber–resin RVE through the aforementioned method. RVE models with varying VC are analyzed in the following sections.

## 4. Simulation of Mechanical Properties of Composite Materials Using RVE Models

### 4.1. Mechanical Modeling of Fibers

CF/PEEK composite materials mainly consist of three components: carbon fibers, the matrix, and the interface. Under transverse loading, the matrix mainly bears the applied load, while the carbon fibers undergo minor elastic deformation and are unlikely to fail. Therefore, no damage model is applied to the fibers in this study. Instead, the fibers are modeled as linear elastic and isotropic materials to reduce model complexity and improve computational efficiency. This modeling approach has been widely used and validated in previous studies [33,34]. The T700 fibers have a Young’s modulus of 15 GPa and a Poisson’s ratio ($\nu_{12}$) of 0.2. The mechanical behavior of the polymer matrix and cohesive elements is thoroughly described below.

### 4.2. Elasto-Plastic Modeling of the Resin

Owing to the high sensitivity of the polymer matrix to hydrostatic pressure, the Mohr–Coulomb criterion [35] is commonly used to describe its mechanical behavior and predict yielding. According to this criterion, yielding occurs on a specific plane once the shear stress exceeds the combined effect of material cohesive strength and the frictional force along the failure surface. The Mohr–Coulomb criterion is mathematically expressed as follows:

(2) $$\left|\tau \right|=c-\sigma_{n}\tan\varphi$$

where c represents the cohesive yield stress, $\sigma_{n}$ denotes the normal stress acting on the failure surface, and φ indicates the internal friction angle.

Thermoplastic resins typically exhibit high ductility and undergo three stages under tensile loading: linear elastic deformation, plastic hardening, and final failure. Once the applied stress reaches resin yield strength, plastic deformation occurs. As the stress approaches the ultimate strength, damage initiates and gradually propagates. However, the Mohr–Coulomb criterion does not capture the progressive failure behavior of the matrix, which limits its accuracy in simulating the complete fracture process. To overcome this limitation, an extended Drucker–Prager yield criterion is introduced to model the plastic hardening behavior of the resin matrix. This criterion effectively captures the asymmetric mechanical response and progressive damage evolution of the resin matrix under both tensile and compressive loading through the incorporation of damage initiation and failure mechanisms. The extended Drucker–Prager yield criterion is mathematically expressed as follows:

(3) F=t−ptanβ−d=0

where p denotes the hydrostatic stress, *t* represents the yield stress, β indicates the material friction angle based on the Drucker–Prager yield criterion, and d signifies material cohesion. The cohesion d can be determined from the uniaxial compressive yield stress $\sigma_{c}$ using the following formula:

(4) $$d=\left(1-\frac{1}{3}\tan\beta \right)\sigma_{c}$$

The yield stress (*t*) can be calculated using the following equation:

(5) $$t=\frac{1}{2}q\left[1+\frac{1}{k}-\left(1-\frac{1}{k}\right){\left(\frac{r}{q}\right)}^{3}\right]$$

where q represents the Mises equivalent stress, and *k* denotes the ratio of the triaxial tensile yield stress to the triaxial compressive yield stress. At *k* = 1, the criterion reduces to the Mises yield condition, indicating equal tensile and compressive yield stresses. For *k* values between 0.788 and 1, the yield surface exhibits an outward convex shape.

The extended Drucker–Prager criterion is derived from the Mohr–Coulomb criterion. Hence, the material parameters β and d can be converted from those of the Mohr–Coulomb criterion using the following equations:

(6) $$\tan\beta =\frac{3\sin\varphi }{\sqrt{3+\sin^{2}\varphi }}$$

(7) $$\frac{d}{c}=\frac{3\cos\varphi }{\sqrt{3+\sin^{2}\varphi }}$$

The value of φ is determined using the following equation:

(8) $$\sin\varphi =\frac{\sigma_{uc}-\sigma_{ut}}{\sigma_{uc}+\sigma_{ut}}$$

Here, $\sigma_{ut}$ and $\sigma_{uc}$ represent the ultimate tensile strength and compressive strength of the resin, respectively.

Experimental results [36] indicate that polymers exhibit brittle fracture behavior under low-strain uniaxial tension but display yielding and significant plastic deformation under uniaxial compression and pure shear. To capture variations in matrix damage behavior across different stress states, a plasticity-based damage initiation criterion is incorporated alongside the yield criterion. This damage criterion assumes that the equivalent plastic strain (${\bar{\varepsilon }}_{D}^{pl}$) at the onset of damage depends on stress triaxiality (η) and the equivalent plastic strain rate (${\dot{\bar{\varepsilon }}}^{pl}$). Damage is considered to be initiated once the material integration point satisfies the following conditions:

(9) $$\omega_{D}=\int \frac{d{\bar{\varepsilon }}^{pl}}{{\bar{\varepsilon }}_{D}^{pl}\left(\eta ,{\dot{\bar{\varepsilon }}}^{pl}\right)}=1$$

where $\omega_{D}$ denotes a state variable that increases monotonically with the accumulation of plastic deformation in the material. The stress triaxiality η is calculated as η=−p/q. Here, p represents the hydrostatic pressure component of the stress tensor, and q denotes the Mises equivalent stress. Stress triaxiality characterizes the nature of the 3D stress state, with different η values corresponding to distinct loading conditions. For example, η = 1/3 under uniaxial tension, η = −2/3 under uniaxial compression, and η = 0 under pure shear.

**Figure 7:**
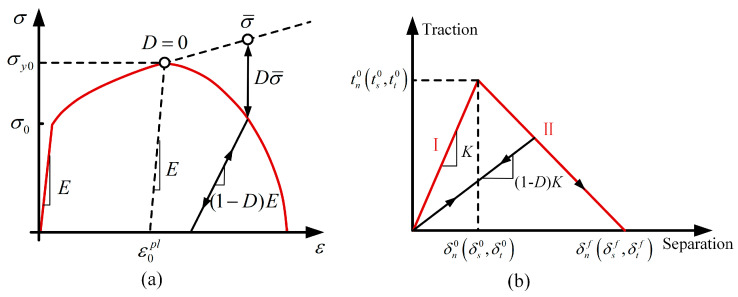
(**a**) Stress–strain curves illustrating the cumulative damage process [37]; (**b**) schematic of the traction–separation law applied in the interfacial cohesive zone model.

Once initial damage occurs, further strain accumulation leads to the progressive development of material damage. This damage evolution follows a gradual failure process. The corresponding stress–strain curves illustrating this cumulative damage behavior are shown in Figure 7a.

In Figure 7a, the solid lines represent the stress–strain response of the material after damage initiation, while the dashed lines indicate the behavior before damage. Here, $\sigma_{y0}$ denotes the yield stress at which damage occurs, ${\bar{\varepsilon }}_{0}^{pl}$ represents the equivalent plastic strain at damage onset (where *D* = 0), and ${\bar{\varepsilon }}_{f}^{pl}$ corresponds to the equivalent plastic strain at complete failure (where *D* = 1). The damage variable *D* controls both the softening of the yield stress and the degradation of the material stiffness. As damage progresses, *D* increases according to the following equation:

(10) $$\dot{D}=\frac{L{\dot{\bar{\varepsilon }}}^{pl}}{{\bar{u}}_{f}^{pl}}=\frac{{\dot{\bar{u}}}^{pl}}{{\bar{u}}_{f}^{pl}}$$

where *L* represents the characteristic length, while ${\bar{\varepsilon }}^{pl}$ and ${\bar{u}}^{pl}$ denote the equivalent plastic strain and equivalent plastic displacement, respectively. Before damage initiation, ${\dot{\bar{u}}}^{pl}=0$; after damage initiation, ${\dot{\bar{u}}}^{pl}=L{\dot{\bar{\varepsilon }}}^{pl}$. The equivalent plastic displacement at failure ${\bar{u}}_{f}^{pl}$ is calculated as ${\bar{u}}_{f}^{pl}=2G_{f}/\sigma_{y0}$. Here, $\sigma_{y0}$ denotes the yield stress at failure, and $G_{f}$ represents the specific fracture energy per unit area. The detailed expression for $G_{f}$ is given as follows:

(11) $$G_{f}=\int_{{\bar{\varepsilon }}_{0}^{pl}}^{{\bar{\varepsilon }}_{f}^{pl}}L\sigma_{y}d{\bar{\varepsilon }}^{pl}=\int_{0}^{{\bar{\mu }}_{f}^{pl}}\sigma_{y}d{\bar{\mu }}^{pl}$$

where ${\bar{\varepsilon }}_{0}^{pl}$ and ${\bar{\varepsilon }}_{f}^{pl}$ represent the equivalent plastic strains at the onset of damage (*D* = 0) and at complete failure (*D* = 1), respectively.

According to Reference [33], the elastic properties of the PEEK matrix are $E_{m}=3.6\;\mathrm{GPa}$ and $\nu_{m}=0.356$, while the parameters of the Mohr–Coulomb model are φ=6° and c=75.03 MPa. Consequently, the tensile strength and compressive strength are $\sigma_{ut}=110\;\mathrm{MPa}$ and $\sigma_{uc}=138\;\mathrm{MPa}$, respectively. The parameter k is then set to 0.927.

### 4.3. Cohesive Zone Model

The constitutive behavior of the cohesive elements is characterized by the bilinear traction–separation law, which defines the relationship between the separation displacement across the element top and bottom surfaces and the corresponding traction vector. The force–displacement response of the cohesive elements governed by this law is shown in Figure 7b.

The curve in Figure 7b consists of two segments. The first stage represents the linear elastic behavior of the interface before reaching its strength limit. The second stage corresponds to the linear degradation of material stiffness after damage initiation. Here, $t_{n}^{0}$ denotes the maximum normal stress the material can withstand, while $t_{s}^{0}$ and $t_{t}^{0}$ represent the maximum shear stresses the material can sustain. $\delta_{n}^{0}$ indicates the displacement in the thickness (normal) direction at the onset of damage, while $\delta_{s}^{0}$ and $\delta_{t}^{0}$ denote the displacements in the two tangential directions at the same stage. Similarly, $\delta_{n}^{f}$ represents the displacement in the thickness (normal) direction at complete failure, while $\delta_{s}^{f}$ and $\delta_{t}^{f}$ correspond to displacements in the two tangential directions at full damage. *K* denotes the slope of the initial linear elastic stage and represents the stiffness of the cohesive element. The constitutive relation governing this linear elastic segment is defined as follows:

(12) $$t_{i}=K_{i}\delta_{i}\left(i=n,s,t\right)$$

where $t_{n}$ and $\delta_{n}$ represent the nominal stress and displacement in the normal direction, respectively, while $t_{s}/t_{t}$ and $\sigma_{s}/\sigma_{t}$ represent the nominal stress and displacement in the shear direction, respectively. Additionally, *K* denotes the interface stiffness.

Once the interfacial tractions reach their respective strength limits, damage initiates within the cohesive element, causing the interface to lose its linear elastic behavior. The cohesive damage model comprises two key criteria: the damage initiation criterion and the damage evolution criterion.

The damage initiation criterion is defined by the quadratic nominal stress criterion. Once the quadratic interaction of the normalized nominal stresses equals 1, damage occurs. This condition is mathematically expressed as follows:

(13) $$\max\left\{\frac{\langle t_{n}\rangle }{t_{n}^{0}},\frac{t_{s}}{t_{s}^{0}},\frac{t_{t}}{t_{t}^{0}}\right\}=1$$

Here, 〈〉 denotes the Macaulay brackets, which yield the enclosed value if positive and zero if negative. This formulation prevents damage development under interface compression.

In cohesive elements, once the initial damage criterion is met, the stiffness of the damaged element gradually degrades with increasing load. In Figure 7b, *K* represents the stiffness matrix of the cohesive element in its undamaged state. Once the stress or strain reaches the initial damage criterion, the stiffness reduces to (1−D)*K*, where D denotes the damage variable quantifying the degree of stiffness degradation. A D value of 0 corresponds to an undamaged element, while D = 1 indicates complete failure. After damage initiation, the damage variable D increases from 0 to 1 with the continuous increase in load.

During material failure, energy is continuously dissipated, commonly known as fracture energy. According to this concept, an energy-based stiffness degradation criterion is established. The displacement at failure is determined by the fracture energy G, which corresponds to the area under the traction–separation curve. The interface parameters used in this study, as reported in Reference [37], are as follows: $K=10^{8}\;\mathrm{GPa}/m$, $t_{n}^{0}=t_{s}^{0}=c=75.03\;\mathrm{MPa}$, and $G=32\;{J/m}^{2}$.

### 4.4. Prediction and Verification of Mechanical Properties

#### 4.4.1. Mesh Sensitivity Analysis

**Figure 8:**
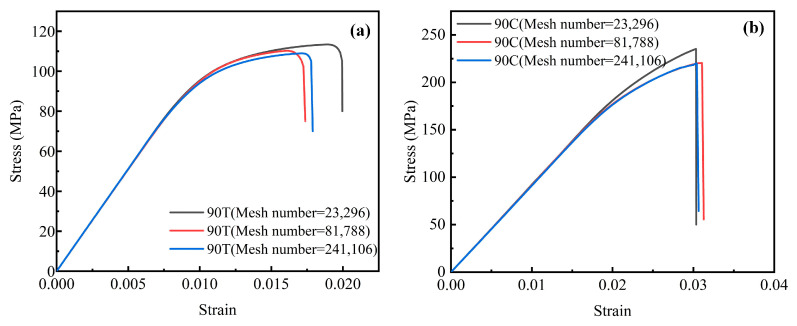
Stress-strain responses of RVEs under (**a**) tensile loading and (**b**) compressive loading at different mesh densities.

The fiber and matrix phases were meshed using 3-node triangular elements (CPE3). To eliminate the effect of mesh density on simulation results, several RVE models with different element counts were constructed. The corresponding stress–strain responses of these models were compared (Figure 8). At an element count of 23,296, the stress–strain responses of these coarser meshes differ noticeably from those of finer meshes, indicating a lack of numerical convergence. In contrast, once the number of elements exceeds 81,788, the predicted mechanical response stabilizes and exhibits minimal variation with further mesh refinement. Therefore, an RVE model comprising 81,788 elements was selected for all subsequent mechanical performance analyses to ensure sufficient accuracy and maintain computational efficiency.

#### 4.4.2. Stress–Strain Curves and Damage Evolution Pathway at Zero Porosity

**Figure 9:**
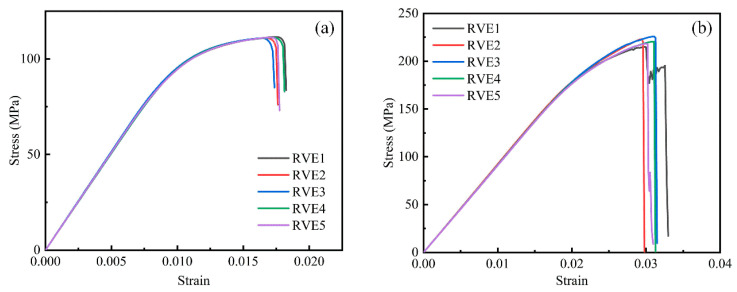
Stress–strain curves of RVEs under (**a**) tensile loading and (**b**) compressive loading.

Figure 9 presents the stress–strain curves of five RVEs under transverse tension and compression at 0% porosity. The results indicate that during the elastic stage, all curves are nearly identical. However, slight deviations are observed after damage initiation. Additionally, tensile fracture occurs at lower strain levels, while compressive failure occurs at higher strain levels.

A distinct yielding stage is observed during tensile fracture, characterized by a gradual increase in stress with increasing strain. For all five RVEs, the average tensile strength is 111.11 MPa, with a low standard deviation of only 0.24%. The average fracture strain is ~1.70%, with a standard deviation of 2.66%.

Regarding compressive properties, the average compressive strength is 220.55 MPa with a standard deviation of 1.89%, while the average fracture strain is ~3.03% with a standard deviation of 2.55%. All deviations are below 5%, indicating the high stability and reliability of the simulation results for tensile strength, compressive strength, and fracture strain.

**Figure 10:**
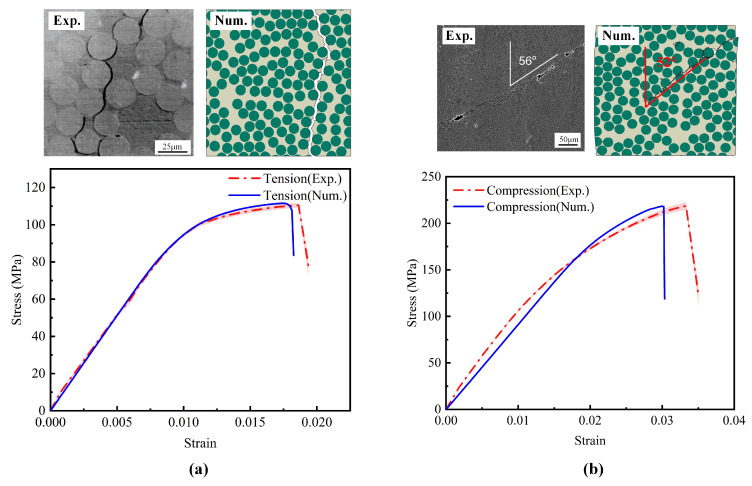
Comparison of experimental and simulated results for composite materials under (**a**) transverse tension loading [38]; (**b**) transverse compressive loading [39].

To validate the effectiveness of the simulation model, Figure 10a,b compare the experimental and simulated results for composite materials under transverse tension and compression. In these figures, the dashed lines represent the experimental data, while the solid lines indicate the simulation predictions. Under tensile loading, the experimental tensile strength is 110.48 ± 1.21 MPa, while the predicted value is 111.50 MPa, corresponding to a relative error of 0.92%. The tensile failure strains are 0.0186 (experimental) and 0.0175 (simulated), yielding an error of 5.91%. Under compressive loading, the experimental compressive strength is 218.74 ± 4.21 MPa, while the predicted value is 220.32 MPa, yielding an error of 0.72%. The compressive failure strains are 0.0333 (experimental) and 0.0300 (simulated), resulting in a 9.91% deviation. Prediction errors for both tensile and compressive strengths are within 5%, while those for failure strains are below 10%. These results indicate that the model accurately predicts both strength and ductility. Overall, the stress–strain curves predicted by the developed model are consistent with the experimental data for both tension and compression tests. Additionally, the fracture morphologies under tensile and compressive loads obtained from finite element simulations are compared with scanning electron microscopy observations [36]. The results indicate a high degree of consistency in failure patterns. A combination of qualitative validation of failure morphologies with the quantitative validation of stress–strain curves confirms that the proposed micromechanical model reliably captures the structure–property relationships under lateral tension and compression conditions.

**Figure 11:**
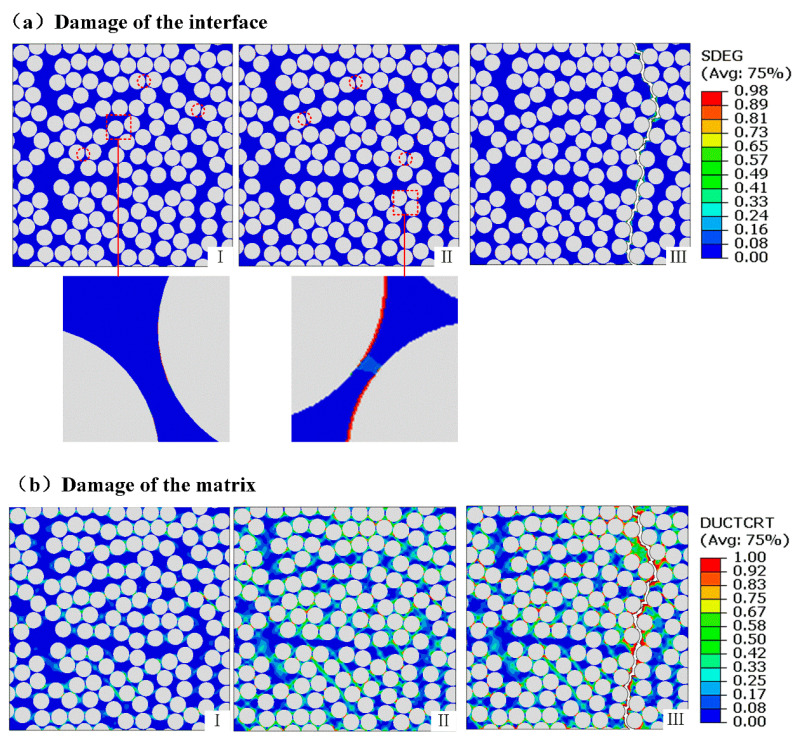
Damage initiation and evolution of RVEs under tensile loading: (I) ε=1.139%; (II) ε=1.675%; (III) ε=1.823%.

Figure 11 illustrates the damage initiation and propagation process in the RVE of the composite materials under tensile loading. The process can be classified into three stages. In the first stage, damage initiates owing to stress concentration from out-of-plane tension, with interfacial damage occurring once the initiation criterion approaches 1.0. At this point, interfacial debonding becomes the dominant damage mechanism, as shown in the magnified view. In the second stage, both the interfacial stiffness degradation variable and the resin ductile damage initiation criterion reach ~1.0. This indicates complete interface failure and the onset of plastic deformation in the adjacent matrix regions. During this stage, plastic damage in the matrix gradually propagates and initially occurs near the debonded interfaces. As loading progresses, debonding extends into additional regions (Figure 11). In the third stage, matrix damage regions merge and connect the previously scattered interfacial debonding zones, causing the complete failure of RVEs. Additionally, the locations of damage initiation (stage 1 in Figure 11) and the subsequent propagation path (stage 3 in Figure 11) may vary owing to localized stress concentrations under tensile loading. The simulation results indicate that the overall failure process is mainly controlled by interfacial debonding and matrix damage.

#### 4.4.3. Stress–Strain Curves at Different Void Levels

**Figure 12:**
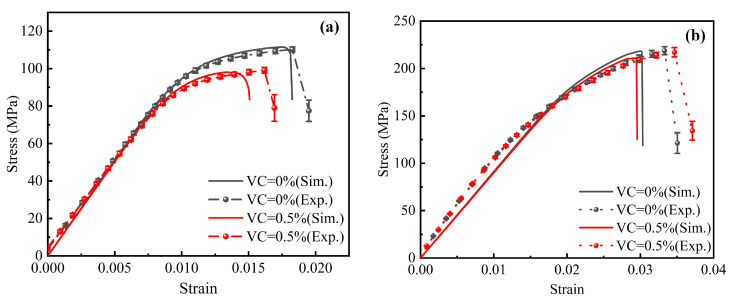
Comparison of mechanical simulation results and experimental data for RVEs with different porosity levels: (**a**) tensile stress–strain curves; (**b**) compressive stress–strain curves.

To further validate the accuracy of the proposed model, the tensile and compressive behavior of the composite material were simulated across varying VC. The simulated results were compared with experimental data. Figure 12a,b present the tensile and compressive stress–strain curves for the composite material at different VC. In these figures, solid lines represent the simulation results, while scatter points correspond to the experimental data.

A comparative analysis reveals that at zero porosity (VC = 0%), the simulation results strongly correlate with the experimental data. This indicates that the model accurately captures the mechanical behavior of the composite material. At a porosity of 0.5%, the model effectively predicts the mechanical response of the material. However, some deviations from the experimental data are observed. These discrepancies may be attributed to the complex distribution of porosity in the experimental samples, variations in the microstructural characteristics, and potential experimental uncertainties.

However, the model successfully characterizes the stress–strain behavior of the composite material across varying porosity levels. This confirms the effectiveness and reliability of the model in simulating the mechanical performance of composite materials.

## 5. Effect of Void Characteristics on the Mechanical Properties of Composite Materials

Previous studies have shown that void defects can severely degrade the service performance of composite materials. However, conventional theoretical models often neglect the mesostructural characteristics of voids, which hinders the investigation of their mesoscopic effects on mechanical properties. Therefore, a comprehensive assessment of the effects of void characteristics on damage behavior is crucial for elucidating composite material performance.

### 5.1. Effect of Void Content

To investigate the effects of void content (VC) on the tensile and compressive strengths of the composite material, a series of RVE models with varying porosity levels were constructed. VC was initially set at 0% and incrementally increased by 0.5% up to a maximum of 2.5%. In the aerospace industry, composite materials typically exhibit a VC below 2%. To minimize the impact of void distribution and size on simulation results, the positions and radii of voids from lower-porosity RVE models were recorded and reused in subsequently developed models. Additional voids were then introduced to achieve the target porosity levels ensuring that voids in each model were sequentially generated using the previous model.

**Figure 13:**
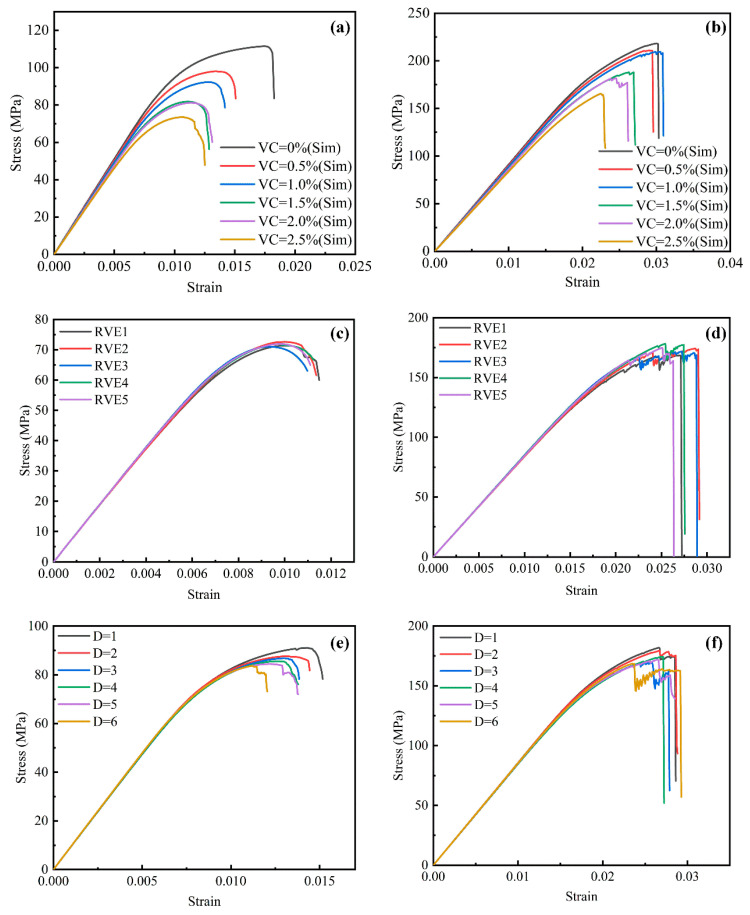
Effect of void characteristics on the transverse mechanical behavior of composite materials: (**a**,**b**) stress–strain curves of RVEs with varying VC under transverse tension and transverse compression, respectively; (**c**,**d**) stress–strain curves of RVEs with different void distributions at 2% porosity under transverse tension and transverse compression; (**e**,**f**) stress–strain curves of RVEs with different pore diameters ranging from 1 to 6 μm under transverse tension and transverse compression.

Figure 13a,b present the stress–strain curves of the RVE model under transverse tensile and compressive loads at different porosity levels (0%, 0.5%, 1.0%, 1.5%, 2.0%, and 2.5%). As porosity increases from 0% to 2.5%, the transverse tensile strength and fracture strain of the composites decrease by 34.1% and 35.3%, respectively. At lower porosity levels (VC = 0% and 0.5%), the material exhibits higher fracture strains, indicating good toughness. In contrast, at higher porosity levels (VC = 1.5%, 2.0%, and 2.5%), failure occurs at lower strain values, reflecting notable ductility loss.

At porosity levels of 1.5% and 2.0%, the stress–strain curves are nearly identical during both the linear and partially nonlinear stages, with only minimal differences in peak stress. This suggests that increasing VC from 1.5% to 2.0% has a limited impact on the transverse elastic modulus. These findings confirm that although VC notably influences the mechanical properties of composite materials, the mesostructural characteristics of voids—such as distribution and size—play a crucial role in determining overall material performance.

Figure 13b shows the stress–strain curves of the RVE model under transverse compressive loading at different porosity levels (0%, 0.5%, 1.0%, 1.5%, 2.0%, and 2.5%). As the porosity increases from 0% to 2.5%, the compressive strength progressively decreases. The highest peak stress occurs at zero porosity (VC = 0%) and significantly decreases as porosity reaches 2.5%. These results indicate that voids compromise the load-bearing capacity of the material, with higher porosity levels causing earlier failure at lower stress thresholds.

At lower porosity levels (0% and 0.5%), the material exhibits noticeable plastic deformation during the nonlinear stage. However, as porosity increases (VC ≥ 1.5%), the plastic deformation region progressively shortens, indicating a transition toward more brittle behavior. Moreover, higher VC leads to a marked reduction in fracture strain, suggesting that material ductility decreases with increasing porosity. This behavior is mainly attributed to stress concentration effects, which promote crack initiation and accelerate crack propagation.

### 5.2. Effect of Void Spatial Distribution

As discussed previously, the mechanical properties of composite materials are influenced by several factors, with VC being an important contributing factor. Other microstructural characteristics of voids—such as their distribution, size, and shape—significantly affect the mechanical behavior of composite materials. To investigate the effect of void distribution on transverse mechanical properties, five RVE models with 2% porosity and varying void arrangements were generated using the random void distribution algorithm described in Section 3.

Figure 13c,d present the stress–strain curves of RVE models with 2% porosity and different void distributions under transverse tensile and compressive loading. During the elastic stage (strain < 0.003), the tensile curves (Figure 13c) are nearly identical, indicating that void distribution has a negligible effect on the transverse tensile elastic modulus. The peak stresses and corresponding failure strains for the five tensile stress–strain curves are 71.42 (0.996%), 72.64 (0.99%), 71.02 (0.93%), 71.91 (0.97%), and 71.94 MPa (0.98%). These results indicate that void distribution has only a minor effect on the transverse tensile strength and failure strain of the composite material.

Figure 13d shows the stress–strain curves of RVE models with different void distributions under transverse compressive loading. The results indicate that void distribution has a minimal effect on both transverse compressive and tensile behavior. Specifically, the five curves display minimal variations during the elastic stage and have similar peak values. These findings suggest that void distribution has a minimal impact on the transverse compressive strength of the composite material.

### 5.3. Effect of Void Size

To investigate the effect of void size on the transverse mechanical properties of composite materials, six sets of RVE models were generated using a random void distribution algorithm. Each model maintained a constant porosity (VC = 2%) but featured varying void diameters. In each RVE model, all voids had the same diameter, which varied between models.

Figure 13e,f present the transverse tensile and compressive stress–strain curves of RVE models with void diameters ranging from 1 to 6 μm at 2% porosity. During the elastic stage, the stress–strain curves for different void diameters (Figure 13e) are nearly identical. This indicates that void diameter has minimal effect on the elastic modulus of the material. At this phase, the overall mechanical response of the composite is mainly controlled by the matrix material, and the effect of void diameter remains negligible. However, in the plastic deformation phase, the effect of void diameter becomes more pronounced. As the void diameter increases from 1 to 6 μm, the peak stresses on the stress–strain curves gradually decrease, leading to a significant reduction in the transverse tensile strength of the material. At smaller void diameters (1 and 2 μm), the peak stress remains relatively high, and the stress–strain curves gradually decline. This indicates enhanced load-bearing capacity during tensile deformation. In contrast, larger void diameters (5 and 6 μm) cause significant reductions in both peak stress and fracture strain. This suggests that larger voids accelerate failure and reduce the overall ductility of the composite.

Figure 13f presents the transverse compressive stress–strain curves of the RVE models with different void diameters ranging from 1 to 6 μm at 2% porosity. Void diameter exhibits a similar effect on transverse compressive and tensile behavior. Specifically, during the elastic stage, the stress–strain curves for different void diameters are nearly identical, indicating that void size has minimal impact on the elastic modulus. However, in the plastic deformation stage, the effect of void diameter becomes more pronounced. As the void diameter increases from 1 to 6 μm, the peak compressive stress gradually decreases, leading to a significant reduction in transverse compressive strength. At smaller void diameters (1 and 2 μm), the peak stress remains relatively high. In contrast, at larger void diameters (5 and 6 μm), both peak stress and fracture strain decrease markedly. This indicates that larger voids accelerate failure under compression.

## 6. Conclusions

This study comprehensively investigates the effects of void characteristics on the mechanical behavior of unidirectional CF/PEEK composites through combined RVE-based modeling and experimental analysis. A novel algorithm is developed to generate RVEs with high fiber volume fraction (≥60%) and embedded void features, in which the void characteristics were statistically derived from micro-CT data.

The results reveal that VC pronouncedly influences the strength and ductility of composite materials. As porosity increases from 0% to 2.5%, the transverse tensile strength and strain at break of the composites significantly decrease by 34.1% and 35.3%, respectively. At higher porosity levels (VC ≥ 1.5%), the material exhibits increased brittleness, characterized by failure at lower strains and a significant reduction in strength. Under compressive loading, voids weaken the load-bearing capacity of the material. In contrast, the void spatial distribution has minimal impact on the elastic response.

Larger void diameters (5–6 μm) accelerate failure and reduce ductility, whereas smaller voids (1–2 μm) maintain superior mechanical performance. These trends indicate that void diameter is a key factor influencing brittleness and failure susceptibility.

In summary, these findings confirm the critical role of void characteristics in composite performance and provide a theoretical basis for designing high-performance composite materials with enhanced mechanical properties for engineering applications.

To reduce modeling complexity and computational cost, voids of varying sizes are simplified using equivalent diameters and uniformly modeled as cylinders based on experimental observations. This approach improves modeling efficiency but compromises simulation accuracy owing to the lack of complete 3D void morphology. Alternatively, a direct FE^2^ method recently proposed by Christof et al. [40] establishes a correlation between macroscopic and microscopic responses, enabling the analysis of local stress evolution under macroscopic loading. Therefore, future research should focus on integrating high-fidelity void reconstruction with FE^2^-based multiscale simulations to improve accuracy and investigate the effect of microscale defects on macroscale performance. 

## Figures and Tables

**Table 1:** Properties of PEEK-CF60 prepreg tape.

	Unit	Value	Standard
Fiber volume fraction	%	60	EN2559
Fiber weight fraction	%	67	EN2559
Tape density	g/cm^3^	1.58	ISO1183
Tape thickness	mm	0.14	——

**Table 2:** Comparison between finite element analysis (FEA) predictions and experimental data.

Mechanical Properties	Experiment	Predicted		Error(%)
		Average	SD	
$\mathrm{Longitudinal}\;\mathrm{modulus},\;E_{11}$ (GPa)	138	137.740	0.002	0.188
$\mathrm{Major}\;\mathrm{Poisson}’s\;\mathrm{ratio},\;\nu_{12}$	0.28	0.287	0.001	2.507
$\mathrm{Transverse}\;\mathrm{modulus},\;E_{22}$ (GPa)	11	9.465	0.027	13.952
$\mathrm{Transverse}\;\mathrm{Poisson}’s\;\mathrm{ratio},\;\nu_{23}$	0.4	0.427	0.002	6.711
In-plane shear modulus, $G_{12}$ (GPa)	5.5	4.918	4.918	10.586

## Data Availability

All original contributions presented in this study are included in the article. Further inquiries can be directed to the corresponding author.
